# Supplementary figures and images for: Understanding brewing trait inheritance in de novo Lager yeast hybrids
Source: mSystems. 2024 Nov 12;9(12):e00762-24. doi: 10.1128/msystems.00762-24 (PMC11651111; doi:10.1128/msystems.00762-24)

A

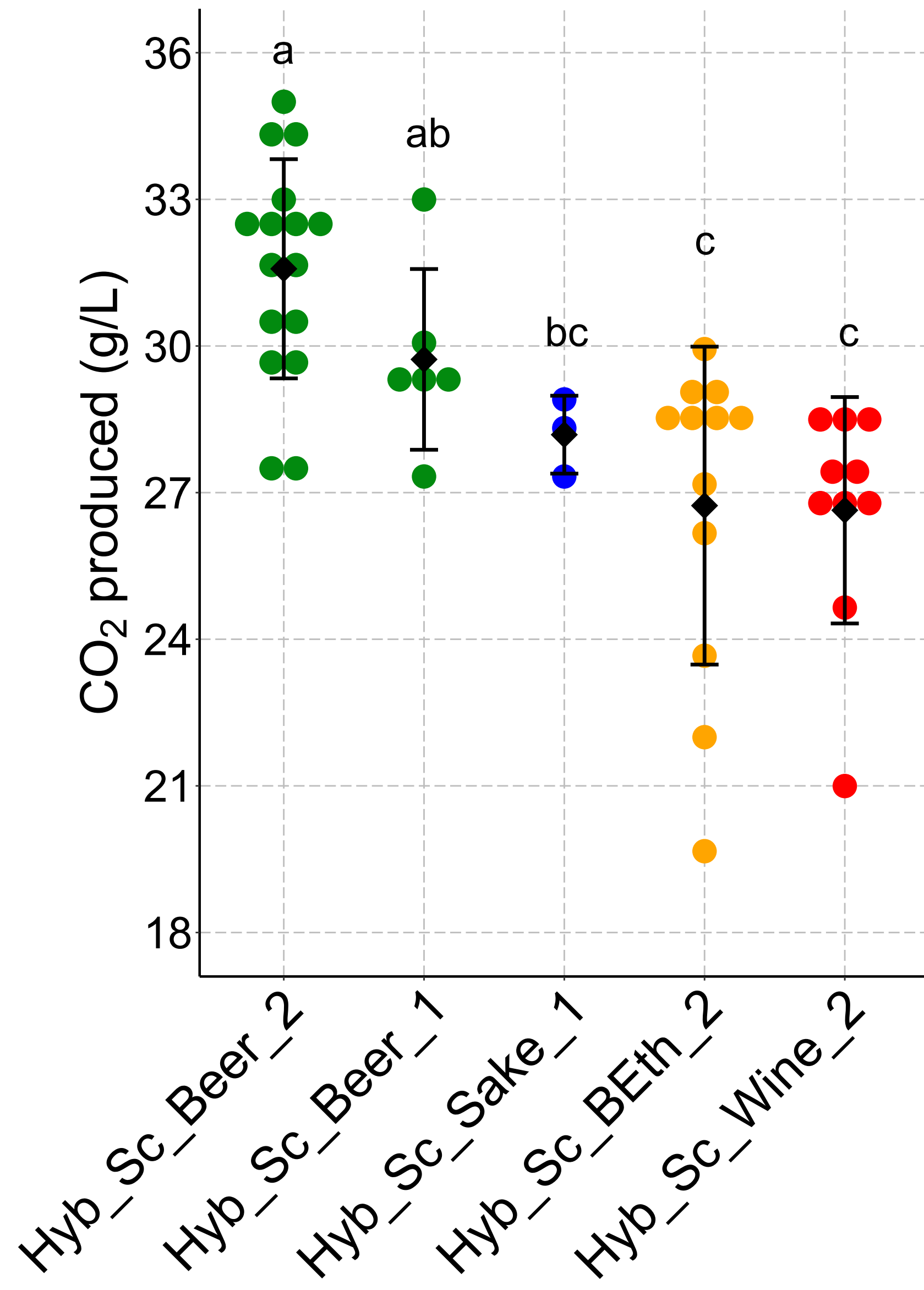

B

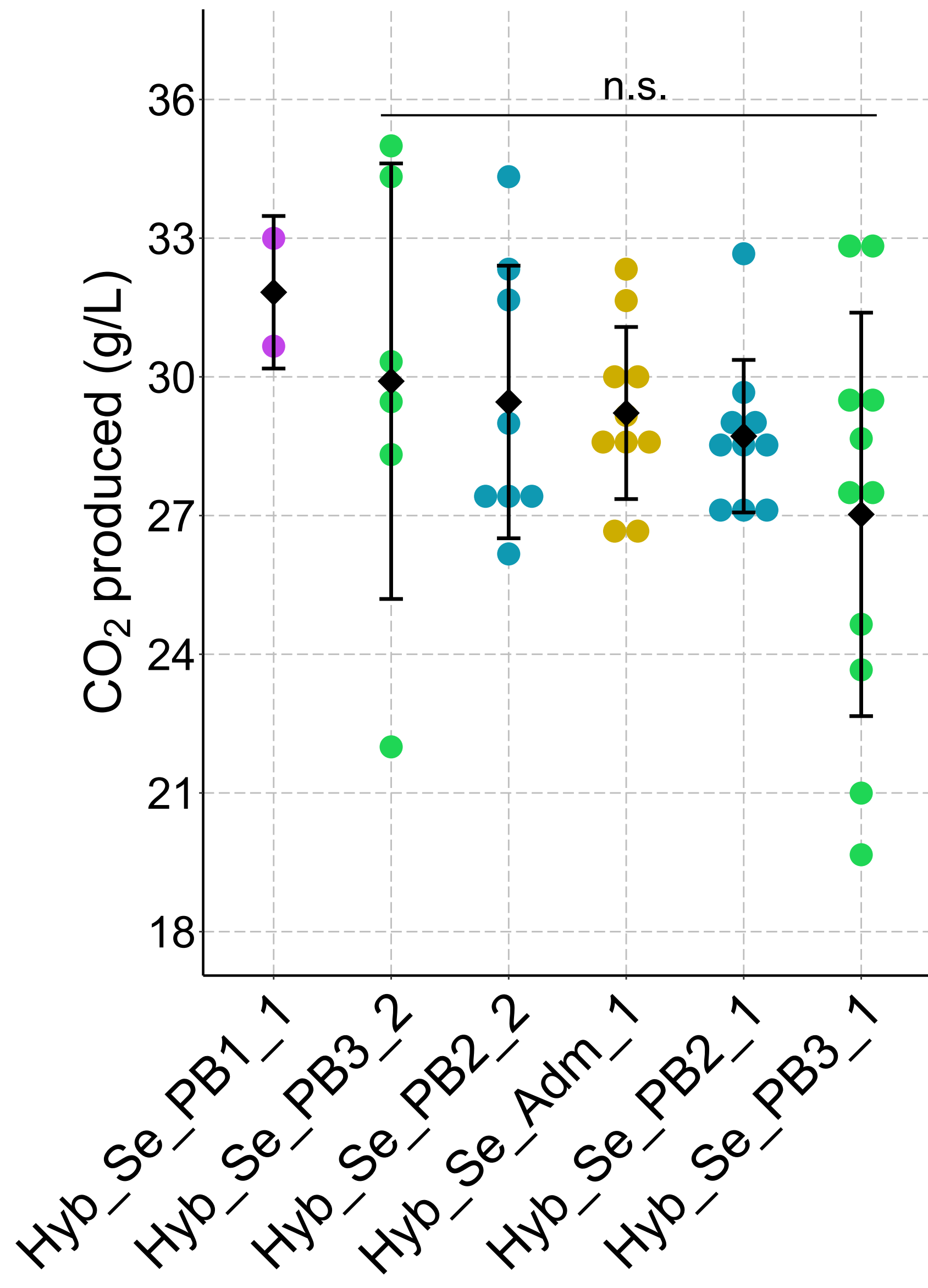

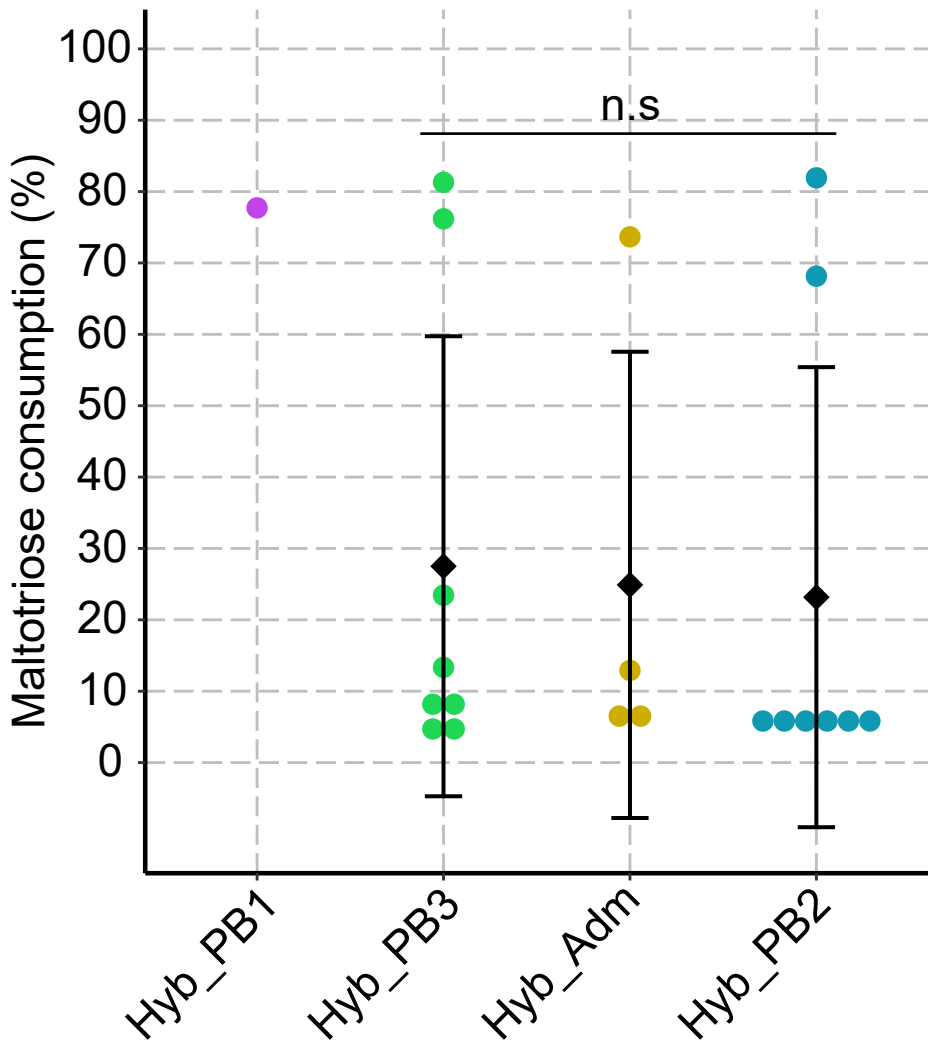

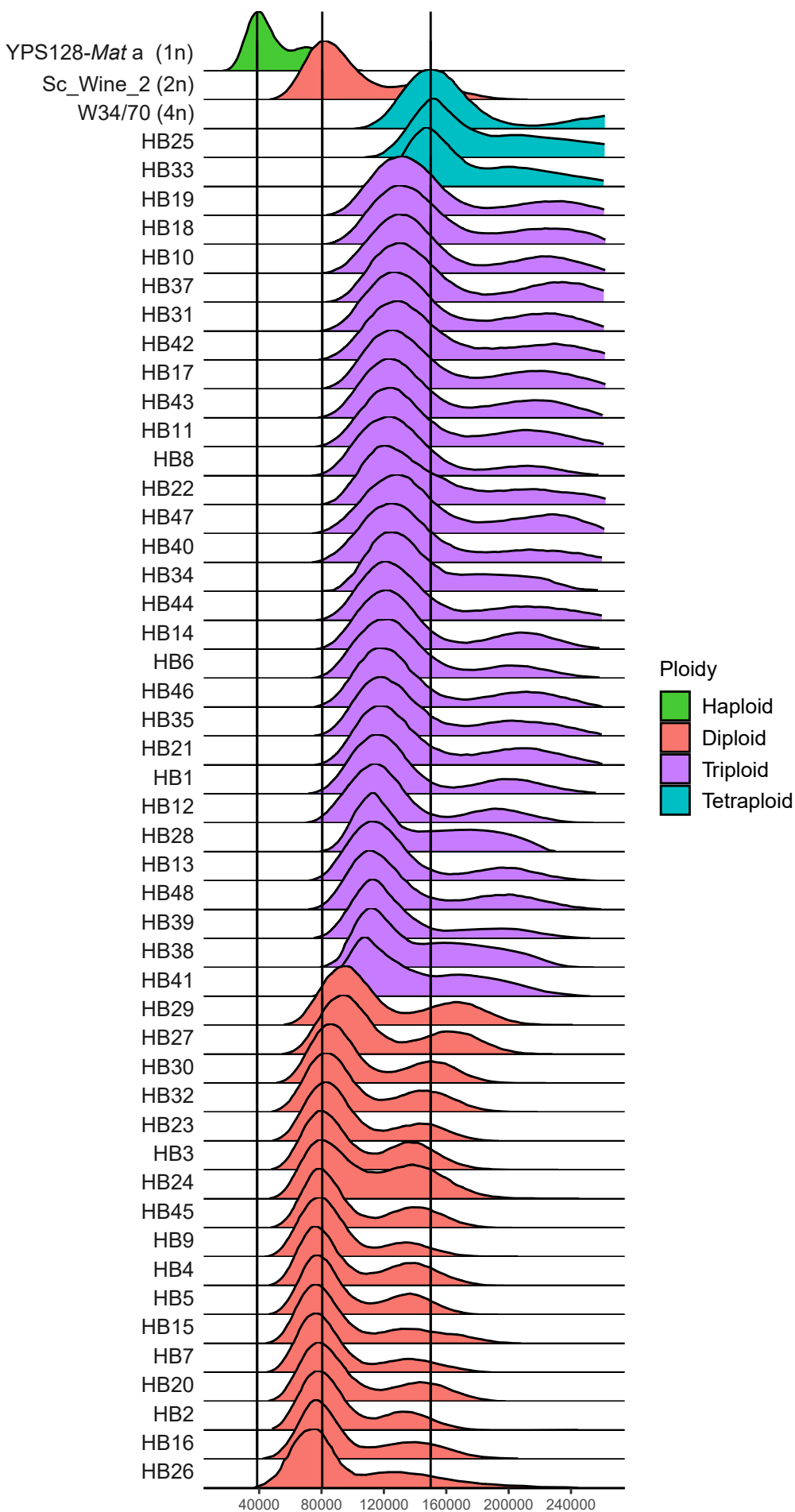

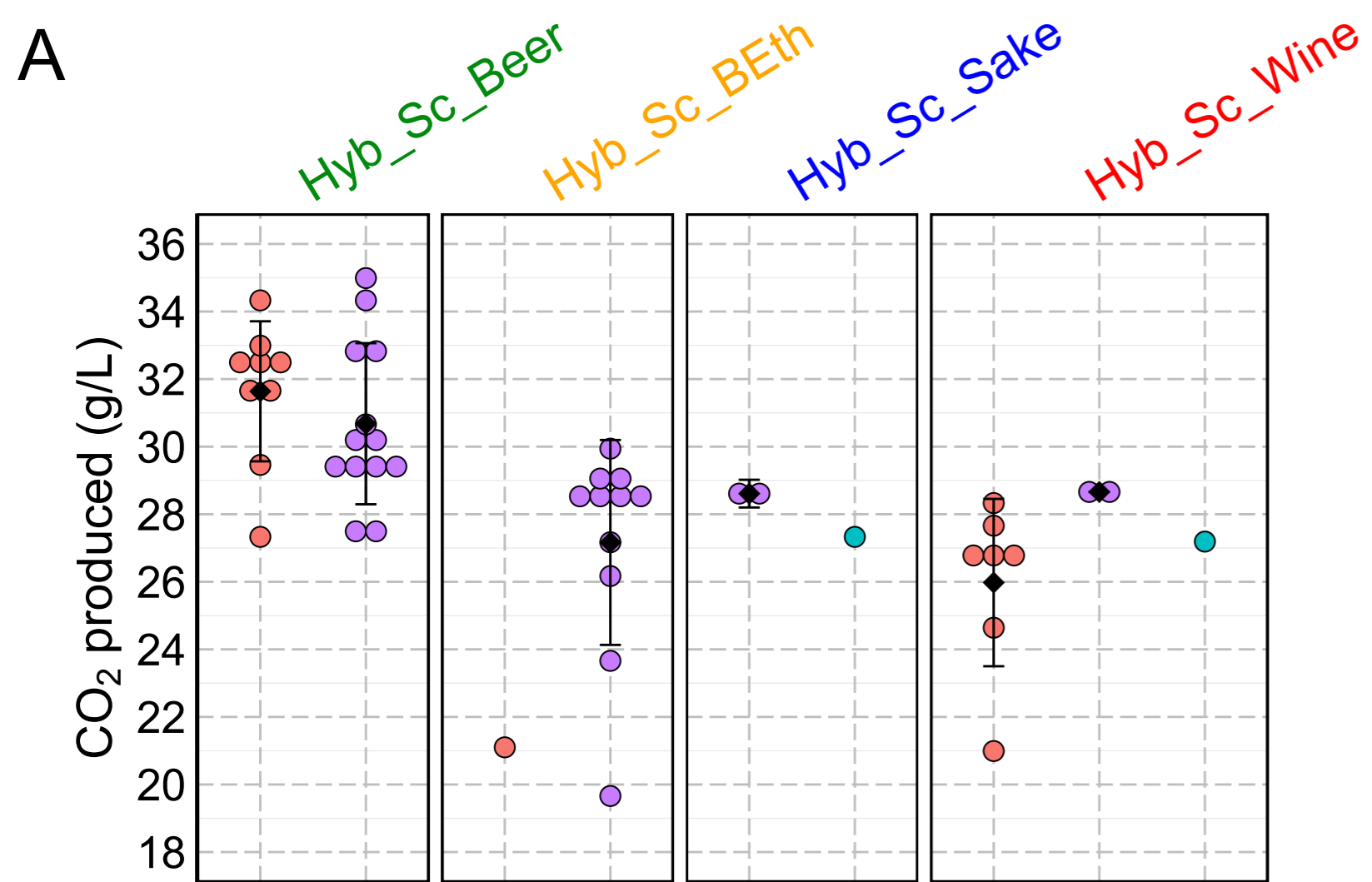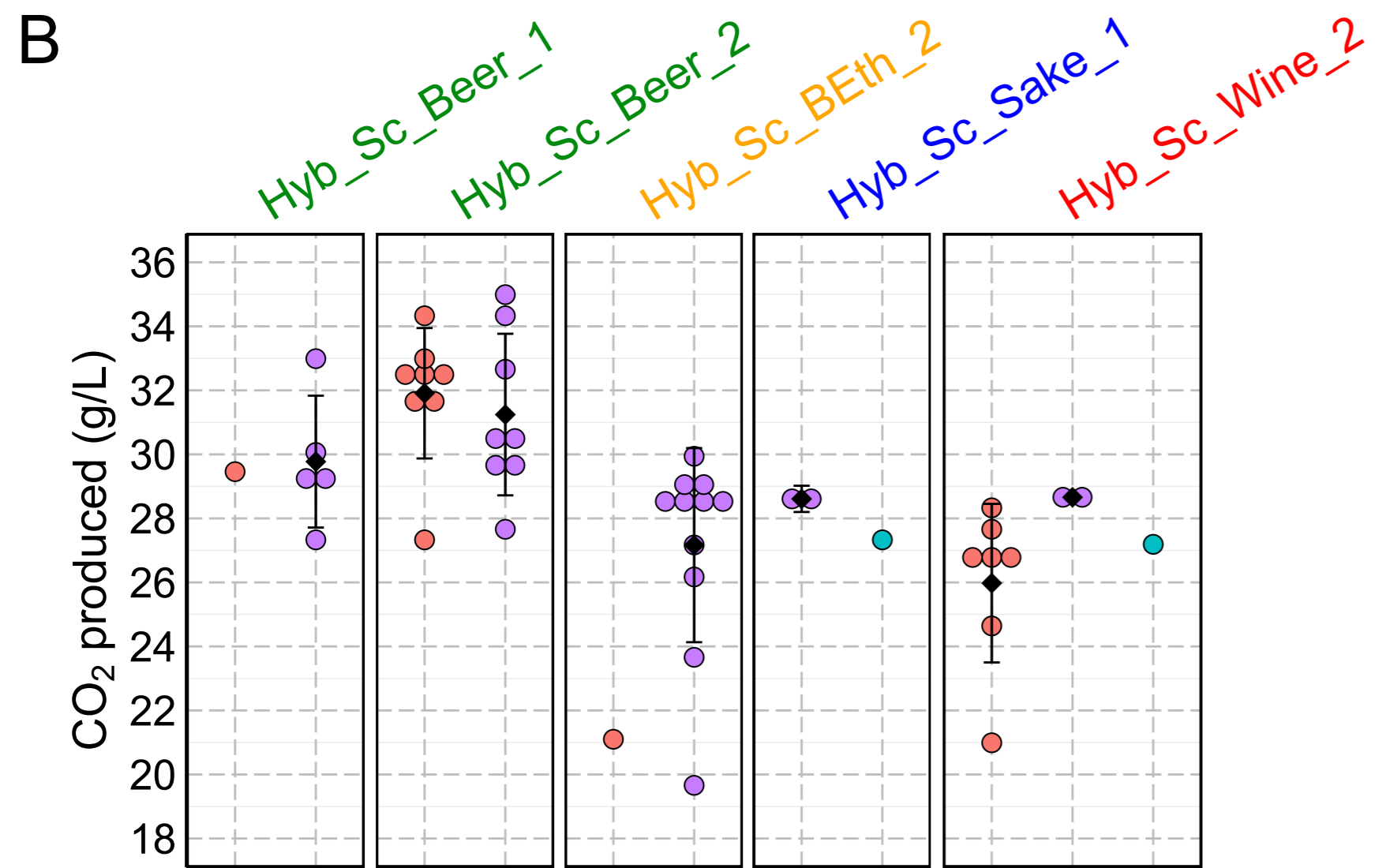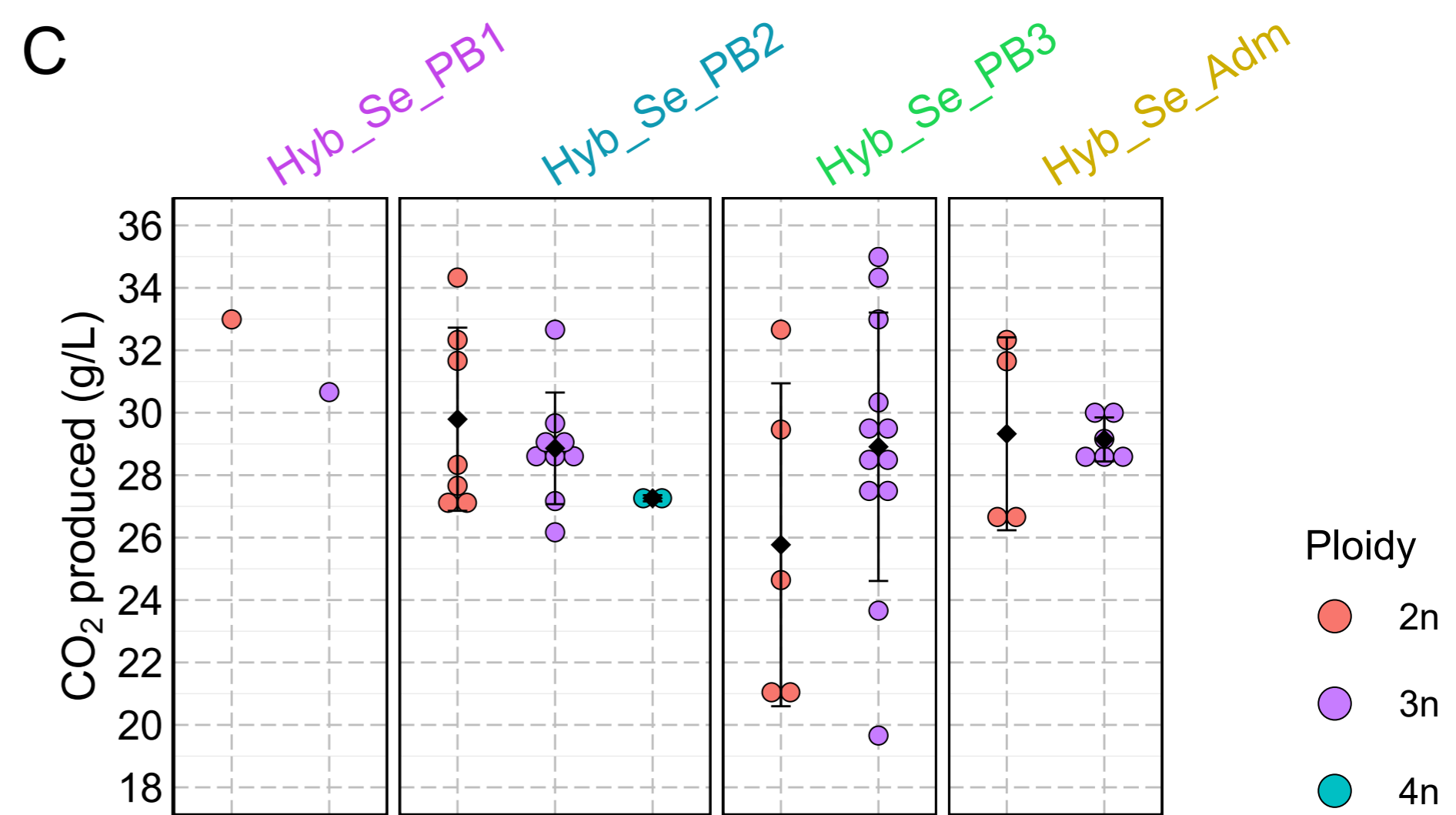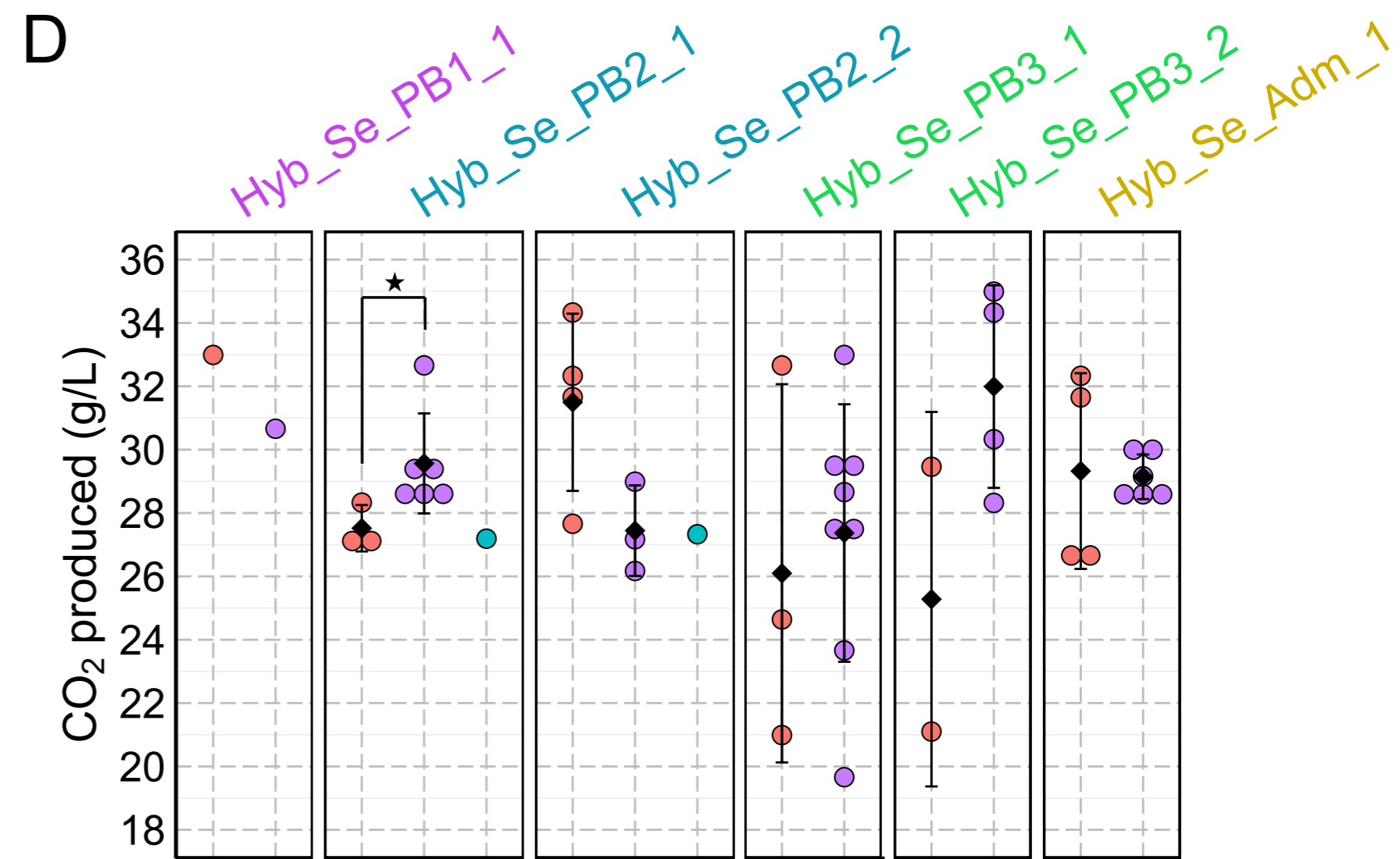

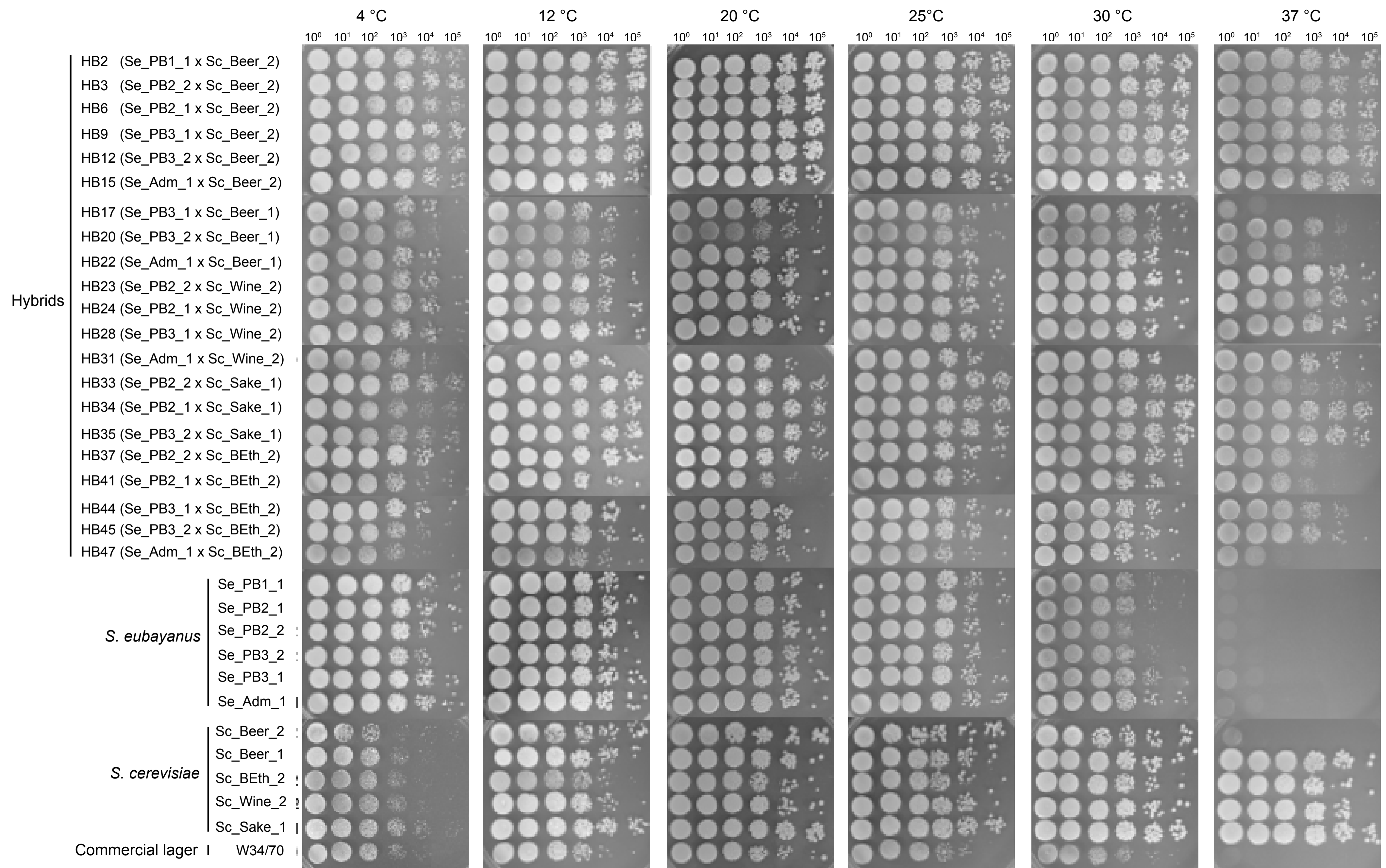

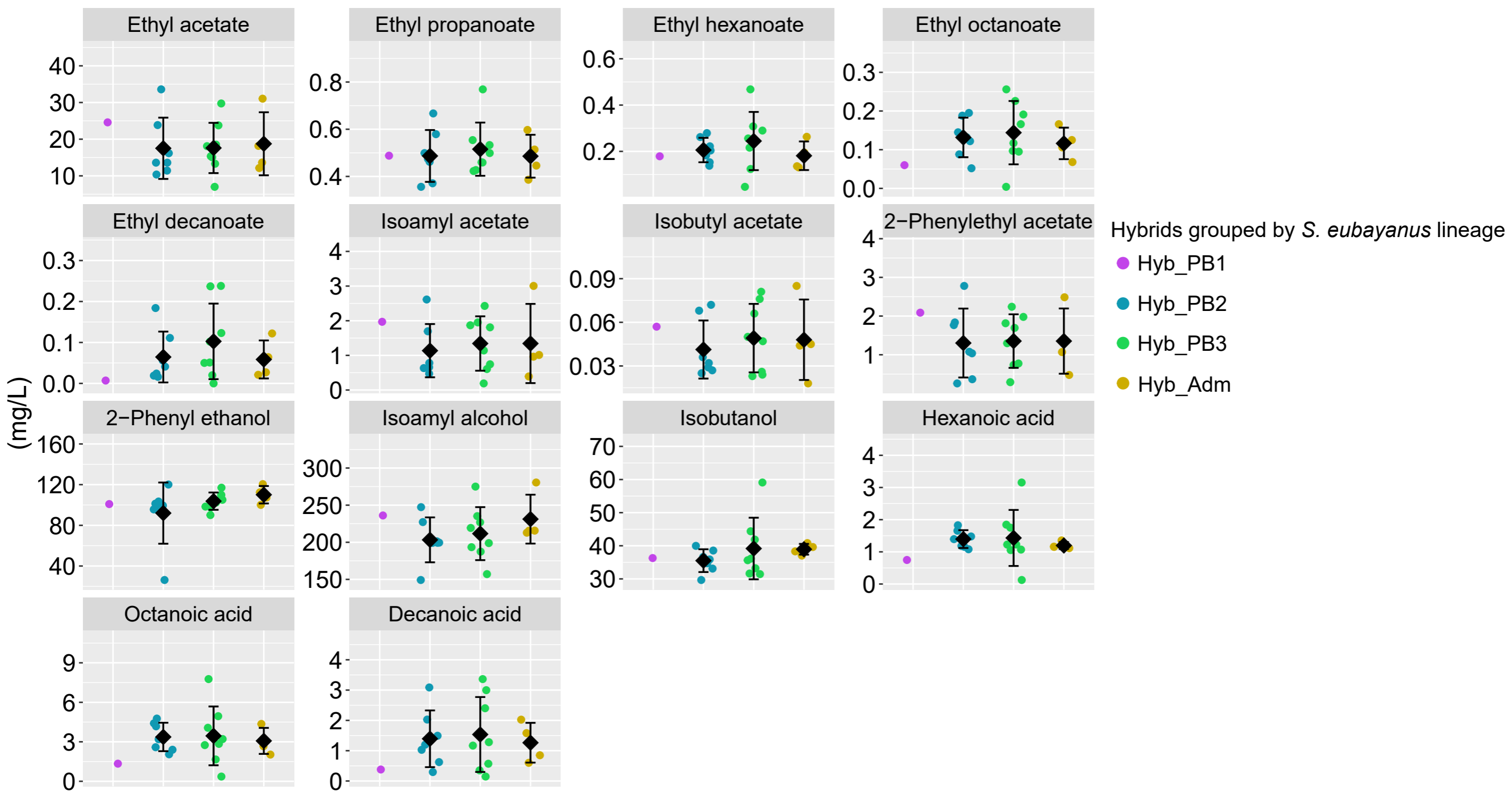

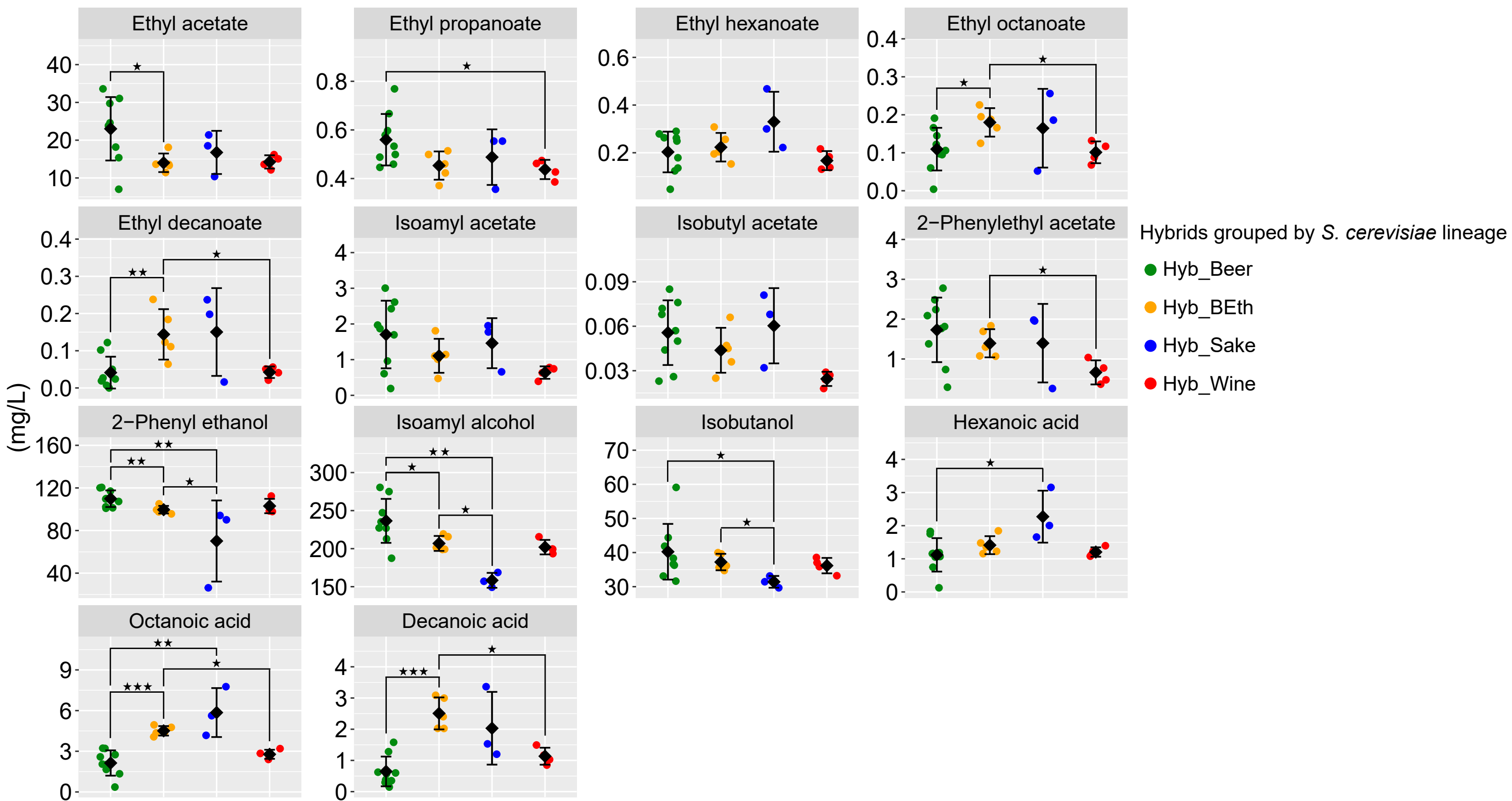

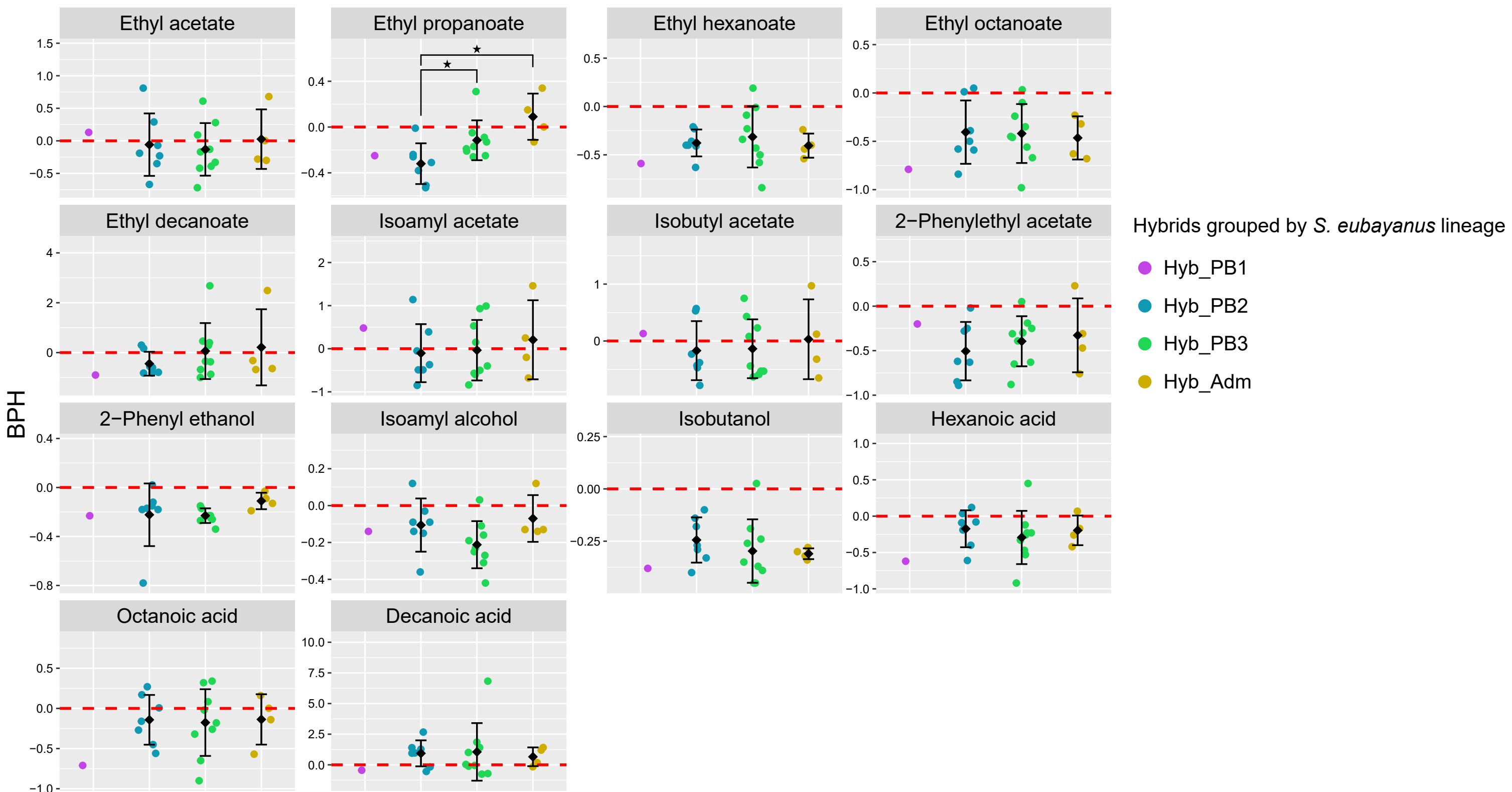

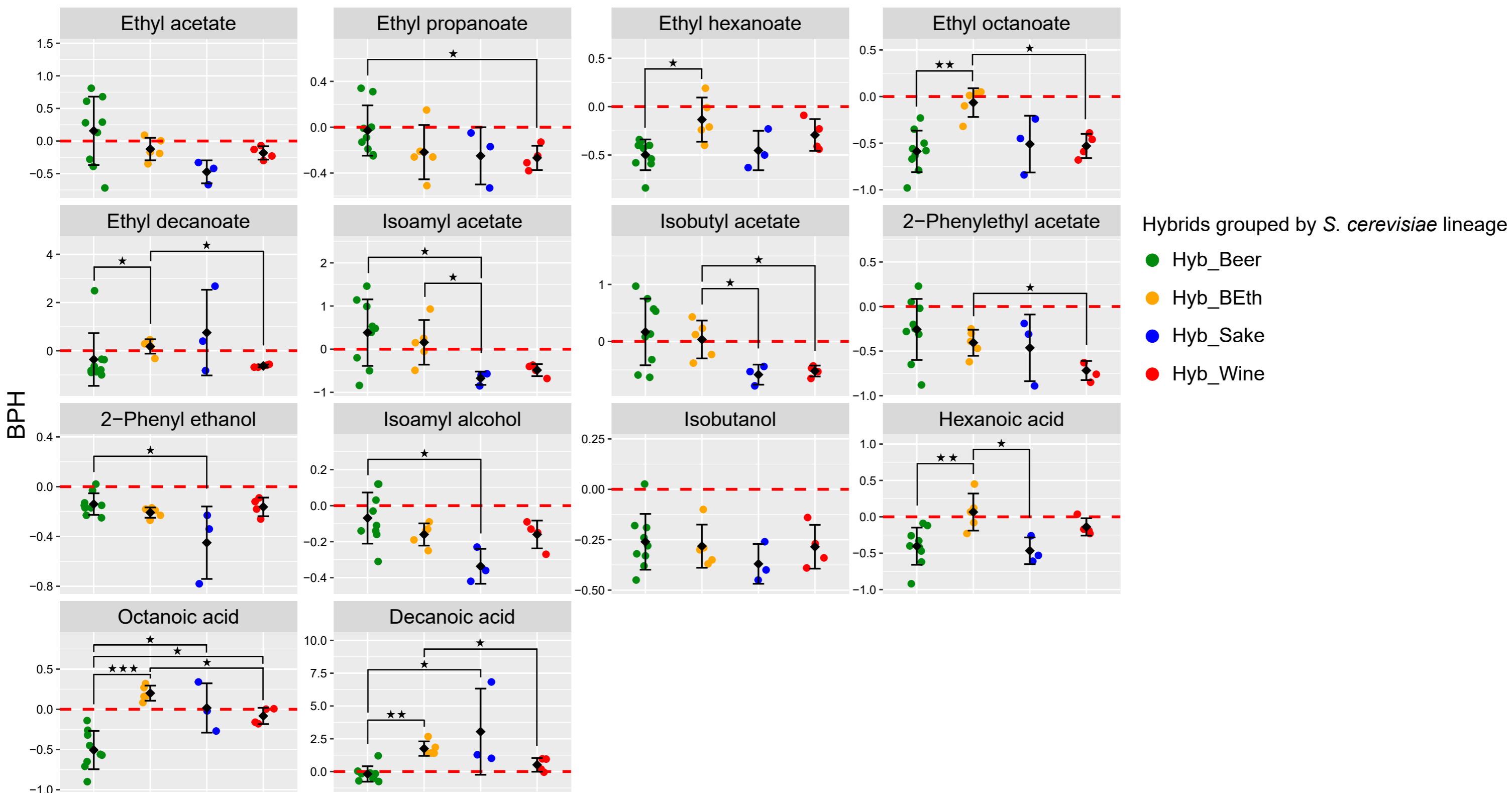

BPH

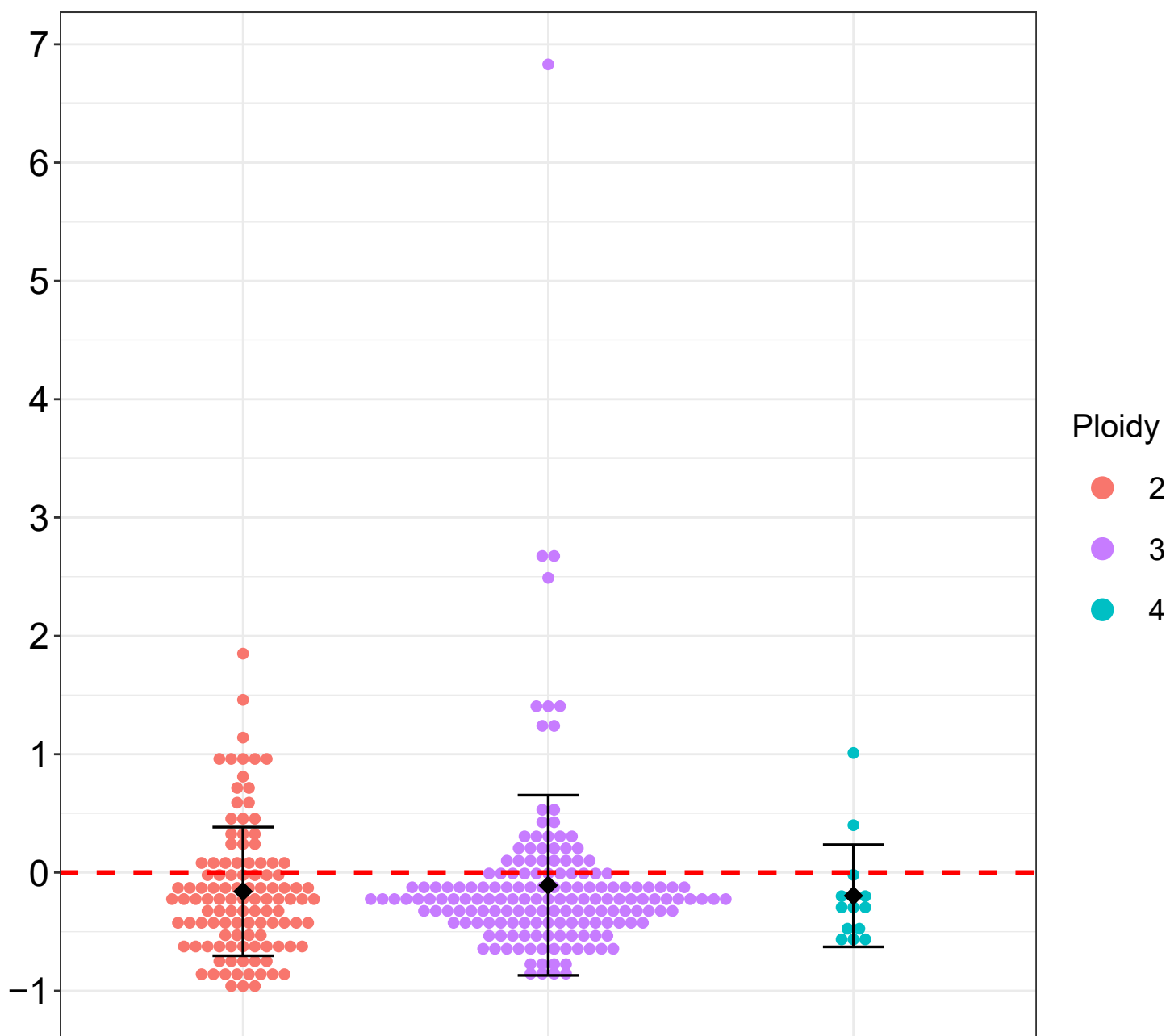

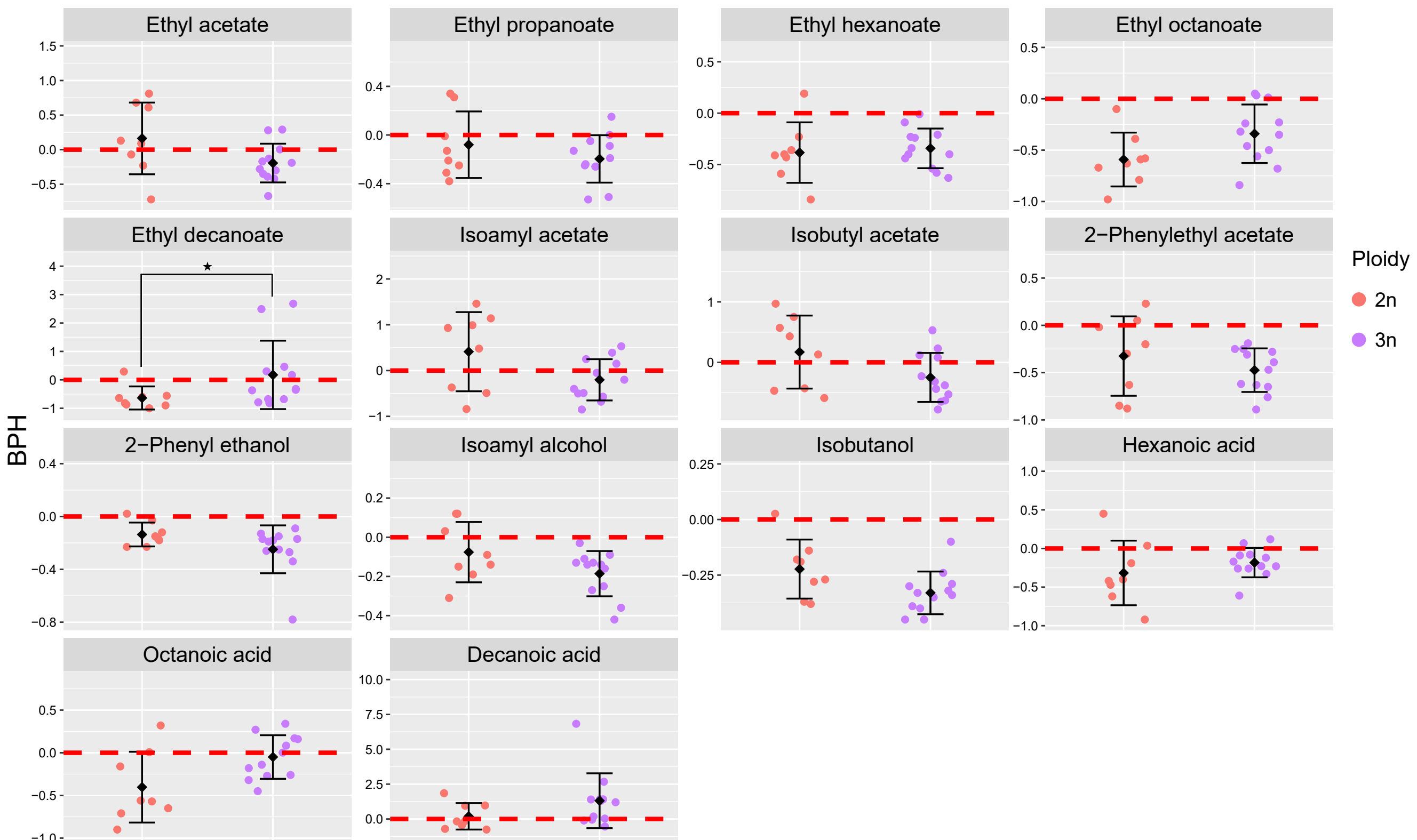

Supplement: Supplemental Figures — Figures S1 to S11. [file msystems.00762-24-s0001.pdf]
