# Supplementary material for: Understanding brewing trait inheritance in de novo Lager yeast hybrids
Source: mSystems. 2024 Nov 12;9(12):e00762-24. doi: 10.1128/msystems.00762-24 (PMC11651111; doi:10.1128/msystems.00762-24)
Supplement: Supplemental legends — Legends to supplemental tables and figures. [file msystems.00762-24-s0002.docx]

**SUPPLEMENTARY INFORMATION**

**FIGURE LEGENDS**

**Figure S1. Fermentative profile of *S. eubayanus* x *S. cerevisae* hybrids depending on the parental strain.** CO_2_ production levels in *de novo* lager hybrids in 12 °P wort depending on the (A) *S. cerevisiae* and (B) *S. eubayanus* parental strains. Lineages are shown in different colours. Diamonds depict mean values. Statistical differences were calculated by using Wilcoxon–Mann–Whitney test, p-value < 0.05. n.s. denotes non-significant differences**.**

**Figure S2. Hybrid’s maltotriose consumption levels under beer wort.** (A) Maltotriose consumption in *de novo* lager hybrids depending on the *S. eubayanus* parental lineages. Diamonds depict mean values. Statistical differences were calculated by using Wilcoxon–Mann–Whitney test, p-value < 0.05. n.s. denotes non-significant differences.

**Figure S3. Ploidy levels of *de novo* hybrids.** Ploidy levels were determined using flow cytometry. Fluorescence curves depicting the DNA content of each strain labeled with propidium iodide are provided. The fluorescence intensity is represented on the x-axis, while the y-axis denotes cell counts, in the y-axis. Approximately 150,000 single cells were used for determining ploidy. Haploid, diploid, and tetraploid standards (*S. cerevisiae* YPS128-*Mat* a, *S. cerevisiae* Sc_Wine_2, and *S. pastorianus* W34/70 strains, respectively) were employed for calibration.

**Figure S4. Fermentative profile of *de novo* lager hybrids sorted by parental lineages and ploidy number.** CO_2_ production levels in *de novo* lager hybrids depending on the (A) *S. cerevisiae* lineage, (B) *S. cerevisiae* strain, (C) *S. eubayanus* lineages and (D) *S. eubayanus* strain. Mean values are depicted by diamonds. Statistical differences were calculated by using Wilcoxon–Mann–Whitney test, p-value < 0.05.

**Figure S5. Hybrid’s temperature tolerance.** A serial dilution assay was conducted on YPD plates with incubation temperatures ranging from 4 to 37 °C. *S. eubayanus* and *S. cerevisiae* parental strains are indicated in blue and red, respectively. Additionally, commercial lager strain W34/70 was included in the assay. Photographs of temperature plate sets were taken at different times (see Methodology for details).

**Figure S6. Volatile compounds production of hybrids depending on the *S. eubayanus* parental lineages.** Beer wort fermentations were conducted for 14 days at 12 °C, and volatile compounds at the end of the fermentation process were determined by GC-FID chromatography. Mean values are depicted by diamonds. No statistical differences were observed in the analysis (Wilcoxon–Mann–Whitney test, p-value > 0.05).

**Figure S7. Volatile compounds production of hybrids depending on the *S. cerevisiae* parental lineages.** Beer wort fermentations were conducted for 14 days at 12 °C, and volatile compounds at the end of the fermentation process were estimated by GC-FID chromatography. Mean values are depicted by diamonds. No statistical differences were observed in the analysis (Wilcoxon–Mann–Whitney test, p-value > 0.05).

**Figure S8. Volatile compound heterosis levels in hybrids depending on the *S. eubayanus* parental lineage.** Heterosis, expressed as best parent heterosis (BPH) was determined relative to the volatile compound production levels of the best parent for the corresponding trait. Red dashed line over the 0 value represents the for the corresponding trait level of the best parent of each hybrid. Mean values are depicted by diamonds. Statistical differences were detected only in ethyl propanoate (Wilcoxon–Mann–Whitney test, p-value < 0.05).

**Figure S9. Volatile compound heterosis in hybrids depending on the *S. cerevisiae* lineage**. Heterosis, expressed as best parent heterosis (BPH) was determined relative to the volatile compound production levels of the best parent for the corresponding trait. Red dashed line over the 0 value represents the volatile compound production levels of the best parent for each hybrid. Mean values are depicted by diamonds. Statistical differences were detected in 13 out of 14 aromas (Wilcoxon–Mann–Whitney test, p-value < 0.05).

**Figure S10. Influence of ploidy on volatile compound heterosis**. Diploid (red), triploid (purple) and tetraploid (sky blue) hybrids are shown. All BPH values for fourteen volatile compounds were included in this analysis. Red dashed line over the 0 value represents the aroma production level of the best parent of each hybrid. Mean values are depicted by diamonds. No statistical differences were observed in the analysis (Wilcoxon–Mann–Whitney test, p-value > 0.05).

**Figure S11. Influence of ploidy on each volatile compound heterosis.** BPH values for fourteen volatile compounds in diploid (red) and triploid (purple) strains are shown. Red dashed line over the 0 value represents the volatile compound levels of the best parent of each hybrid. Mean values are depicted by diamonds. Statistical differences were detected only in ethyl decanoate (Wilcoxon–Mann–Whitney test, p-value < 0.05).

**TABLE LEGENDS**

**Table S1. Strains used in this study.**

**Table S2. Sugars consumption (g/L) after 14 days under beer wort fermentation**. R= replicates, SD= standar deviation. A. *S. eubayanus* strains, B. *S. cerevisiae***.**

**Table S3. Hybridization success rate.**

**Table S4. *S. cerevisiae* x *S. eubayanus* hybrids.**

**Table S5. Fermentative capacity of hybrids under beer wort.** R= replicates, SD= standar deviation.

**Table S6. Sugars consumption of hybrids after 14 days in beer wort fermentation.** R= replicates, SD= standar deviation.

**Table S7. Maltoriose consumption in hybrids after 14 days in beer wort fermentation**. SD= standar deviation.

**Table S8. Ploidy levels in hybrids determined by flow cytometry.**

**Table S9. Area Under the Curve (AUC) in hybrids grown under different environmental conditions.**

**Table S10. Volatile compounds production in hybrids and parental strains.**

**Table S11. Volatile compounds production in hybrids depending on the parental lineage.**

**Table S12. 4-VG production in hybrids and parental strains.**

**Table S13. Best patent heterosis (BPH) levels in hybrids.**

**Table S14. Best parent heterosis (BPH) in volatile compounds levels depending on the parental lineage.**

**Table S15. RNA-seq results. Design = HB6 vs HB41.**
